# The efficacy of Tripterygium Glycosides in the treatment of Chinese patients with thyroid-associated orbitopathy: a systematic review and meta-analysis

**DOI:** 10.3389/fphar.2024.1433791

**Published:** 2025-01-06

**Authors:** Mingzhe Li, Bingchen Wei, Tianshu Gao, Chenghan Gao

**Affiliations:** ^1^ Internal Medicine Department, The Third Affiliated Hospital of Liaoning University of Traditional Chinese Medicine, Shenyang, China; ^2^ The First Clinical College, Liaoning University of Traditional Chinese Medicine, Shenyang, China; ^3^ The First Affiliated Hospital, Liaoning University of Traditional Chinese Medicine, Shenyang, China

**Keywords:** hyperthyroidism, thyrotoxicosis, thyroid-associated orbitopathy, Tripterygium Glycosides, meta-analysis

## Abstract

**Objective:**

This study aims to conduct a systematic review of the effectiveness and safety of Tripterygium Glycosides interventions in the treatment of Chinese patients with thyroid-associated orbitopathy (TAO).

**Methods:**

A literature search was conducted using PubMed for English sources, and the CNKI, Chinese Biomedical Database, Wanfang Database, and VIP Database for Chinese sources. The search period extended from the beginning of the databases’ creation to Dec. 2023. The keywords used in the search were hyperthyroidism, thyroid-related immune orbitopathy (TRIO), ophthalmopathy, and Tripterygium Glycosides. Various combinations of search terms were used, depending on the database being queried. All the trials included in the study were clinical randomized controlled trials (RCTs).

**Results:**

33 RCTs or quasi-RCTs that met the inclusion criteria were included. The meta-analysis included 27 RCTs. 6 RCTs were excluded from the analysis due to the absence of a control group, but they were still included in the systematic review. 27 RCTs or quasi-RCTs involving 2,134 patients were included in the meta-analysis. The TRIO patients in the treatment group received Tripterygium Glycosides in combination with Thiamazole, Prednisone, Levothyroxine sodium, or a combination of these medications. While the TRIO patients in the control group were treated with Thiamazole, Prednisone, Levothyroxine sodium, or a combination of these treatments, the meta-analysis results show that the overall effectiveness rate of the treatment group and the control group was *P* = 0.05, I^2^ = 0.33 < 0.5 [MD = 4.45, 95% CI (3.31, 5.99), *P* < 0.00001]. The former was significantly superior to the latter. At the same time, a risk assessment was conducted for the study of the 2 groups. The former was significantly superior to the latter. Furthermore, the clinical effectiveness rate of eyeball prominence was *P* < 0.00001, I^2^ = 0.98 > 0.5 [MD = 2.40, 95% CI (2.28, 2.51), *P* < 0.00001]. The clinical effectiveness rate of CAS score was *P* < 0.00001, I^2^ = 0.89 > 0.5 [MD = 1.68, 95% CI (1.50, 1.85), *P* < 0.00001]. The clinical effectiveness rate of FT_3_ was *P* < 0.00001, I^2^ = 0.98 > 0.5 [MD = 0.95, 95% CI (0.81, 1.08), *P* < 0.00001], the clinical effectiveness rate of FT_4_ was *P* < 0.00001, I^2^ = 0.95 > 0.5 [MD = 2.12, 95% CI (1.99, 2.25), *P* < 0.00001], and the clinical effectiveness rate of TSH was *P* < 0.00001, I^2^ = 0.89 > 0.5 [MD = −0.19, 95% CI (−0.21, −0.17), *P* < 0.00001].

**Conclusion:**

The experience with the treatment of TAO using Tripterygium Glycosides was promising. The existing evidence suggests that treatment with Tripterygium Glycosides may be more effective in enhancing the response rate, quality of life, and FT_3_ levels compared to treatment with Prednisone, Levothyroxine sodium, and/or Thiamazole alone.

## 1 Introduction

Thyroid-associated ophthalmopathy (TAO) is a group of autoimmune diseases involving orbital and periocular tissues associated with genetic, environmental, and immunologic factors, with the highest incidence of orbital disease. The pathogenesis of the disease is complex, with the majority of patients suffering from Graves’ disease (GD), which has a prevalence of up to 40%. Moreover, in 80% of patients experiencing both hyperthyroidism and ophthalmopathy, the clinical symptoms progress rapidly within 2 years of disease onset, forming a vicious cycle (Ying, 2009). Graves’ ophthalmopathy is also known as thyroid eye disease (TED), thyroid-associated orbitopathy (TAO), and Graves’ orbitopathy (GO) (Zhang and Wang, 2010; Webb et al., 2010). Tripterygium wilfordii is the Chinese herbalanti-inflammatory immunomodulator, which is the first studied andused in China, known as the “Chinese herbal hormone”. It has thefunctions of promoting blood circulation and collateralization, dispelling wind and dehumidification,detumescence and pain, detoxification, anti-inflammatory etc. Extract of tripterygium wilfordii is often used in the treatment of autoimmune diseases. There is a large number of clinical studies having found thattripterygium wilfordii can be used in the treatment ofthyroid-associated ophthalmopathy (Li, 1987). Currently, the use of Tripterygium and its extracts for treating hyperthyroidism exophthalmos is gaining clinical attention. Comprehensive analysis and evaluation of RCTs on TAO with Tripterygium Glycosides were carried out in this paper according to principles of evidence-based medicine. A meta-analysis was conducted to provide objective and accurate evidence, and to assess the effectiveness of Tripterygium Glycosides in treating hyperthyroidism exophthalmos. The aim was to offer guidance and a foundation for the clinical use of this medication.

Tripterygium wilfordii is a perennial vine species in the Celastraceae family, extensively utilized in traditional Chinese medicine for the treatment of autoimmune and inflammatory diseases. According to the *Compendium of Materia Medica*, Tripterygium wilfordii is documented as a treatment for conditions such as swelling, edema, accumulation, yellow and white pox, long-term incurable malaria, constipation, leprosy, and falls.

The protocol of this network meta-analysis was registered in PROSPERO with ID CRD42021247873. We present the following article in accordance with the PRISMA reporting checklist (available at https://dx.doi.org/10.21037/apm-21-1307).

## 2 Methods

### 2.1 Literature sources and search

The publications utilized in the meta-analysis were identified through searches of the China National Knowledge Infrastructure (CNKI), PubMed, Web of Science, Wanfang Database, VIP Database, and EMBASE. The search period extended from the inception of the databases’ construction to December 2023, and the search was conducted in Chinese or English. The key words used in the search were “Hyperthyroidism”, “Thyroid related immune orbitopathy” or “TRIO”, “Ophthalmopathy” or “Tripterygium Glycosides”, “Tripterygium, Tripterygium wilfordii”, “Tripterygium wilfordii Hook f.“, “Tripterygium wilfordii multiglycoside”. Different combinations of search terms were used, depending on the selected database. The selected publications were clinical trials published in medical journals. 2 reviewers independently evaluated English and Chinese literature for inclusion. Any disagreements were resolved through discussion.

#### 2.1.1 Literature selection

Research on the types of RCTs (RCT or Controlled Clinical Trial, CCT) for the treatment of TAO, regardless of whether blinded or not.

Diagnose standard according to the *Diagnosis of clinical diseases based on the improvement of the standard* (Ministry of health of the people’s Liberation Army General Logistics Department, 1987): (1) typical ocular symptoms; (2) with hyperthyroidism or a history of hyperthyroidism; and (3) excluding other similar diseases.

Exclusion criteria: (1) myopia; (2) orbital inflammatory pseudotumor; (3) carotid-cavernous sinus fistula or dural artery cavernous sinus; (4) extraocular muscle lymphatic tumor; (5) primary orbital tumor; (6) ocular metastasis; and (7) intracranial tumors and other diseases.

TAO classification according to Wilmar’s simple classification standard. Grade Ⅰ (mild): eyeball prominence <18mm, with upper eyelid retraction, gaze, eyelid, and conjunctival edema; Grade Ⅱ (moderate): eyeball prominence is 18–20 mm, with ocular involvement; Grade Ⅲ (severe): eyeball prominence >20 mm, with corneal involvement and vision disorders (Ministry of Health PRC, 1993).

#### 2.1.2 Literature extraction

Studies were excluded if they were: (1) animal experiments; (2) clinical trials from which no relevant data could be extracted; (3) repeatedly published studies; (4) studies involving patients with serious mental disorders or dementia; (5) studies involving patients with serious systemic symptoms that may significantly affect their ability to perform daily living activities, including syncope or coma, seizure-like headache, and cachexia; and (6) studies involving pregnant or breastfeeding women.

All trials included in the analysis were extracted by two reviewers. Once completed, any disagreements regarding data extraction and study evaluation were resolved through discussion with the third reviewer. All the trials included in the analysis contain information on study design, patient characteristics, and medication use.

### 2.2 Clinical efficacy

Clinical efficacy judgement (Wiersinga and Prummel, 2002; Gu et al., 2003): Cure is defined as the disappearance of eye symptoms, obvious retraction of the eye, protrusion of the eyeballs <18 mm, or a reduction in protrusion by >3 mm. The treatment was significantly effective as the eye symptoms disappeared, but the reduction in exophthalmos >2 mm. The degree of reduction in exophthalmos is effective, ranging from 1–2 mm. Invalid: The degree of exophthalmos showed no obvious change, or exophthalmos reduced by <1 mm.

### 2.3 Quality assessment

Following the quality assessment standard recommended by the Cochrane Review Handbook 5.0 (Higgins et al., 2023). The bias risk assessment tool involved six aspects: (1) random distribution method; (2) concealment of allocation decisions; (3) blinding of research subjects, operators of the therapeutic plan, or those measuring the results; (4) result integrity; (5) selective presentation of study findings; and (6) other potential sources of bias.

Each research result was evaluated based on the six aspects mentioned above and categorized as “YES” (low-degree bias), “NO” (high-degree bias), or “unclear” (lacking relevant information or uncertain bias condition). Two evaluators cross-verified the quality assessment results of the inclusive trials, and any differences in opinions were resolved through discussion or third-party arbitration.

### 2.4 Statistical analysis

Meta-analysis was performed using the Rev Man software (Version 5.3) from The Cochrane Collaboration website. First, we performed clinical heterogeneity and methodological heterogeneity analyses for all the trials included. Statistical heterogeneity was evaluated by the Chi-squared (χ^2^) test and heterogeneity were considered present if *P* ≤ 0.10. A quantitative assessment of heterogeneity was performed using I2 where I2 > 50% indicated high heterogeneity among study results. Study results were pooled for analysis using a fixed effects model when there was no statistical heterogeneity or using a random effects model when statistical heterogeneity was detected. Outcome indicators were presented as mean differences (MD) along with their 95% confidence intervals (95% CI) for continuous variables, and as odds ratios (OR) with 95% confidence intervals (CI) for categorical variables. For hypothesis testing, the U test was used and the results were presented as Z and P values. The differences in the efficacy between interventions were considered statistically significant if *P* ≤ 0.05. The results of hypothesis testing are presented in a forest plot.

## 3 Results

### 3.1 Identified studies and characteristics

**FIGURE 1:**
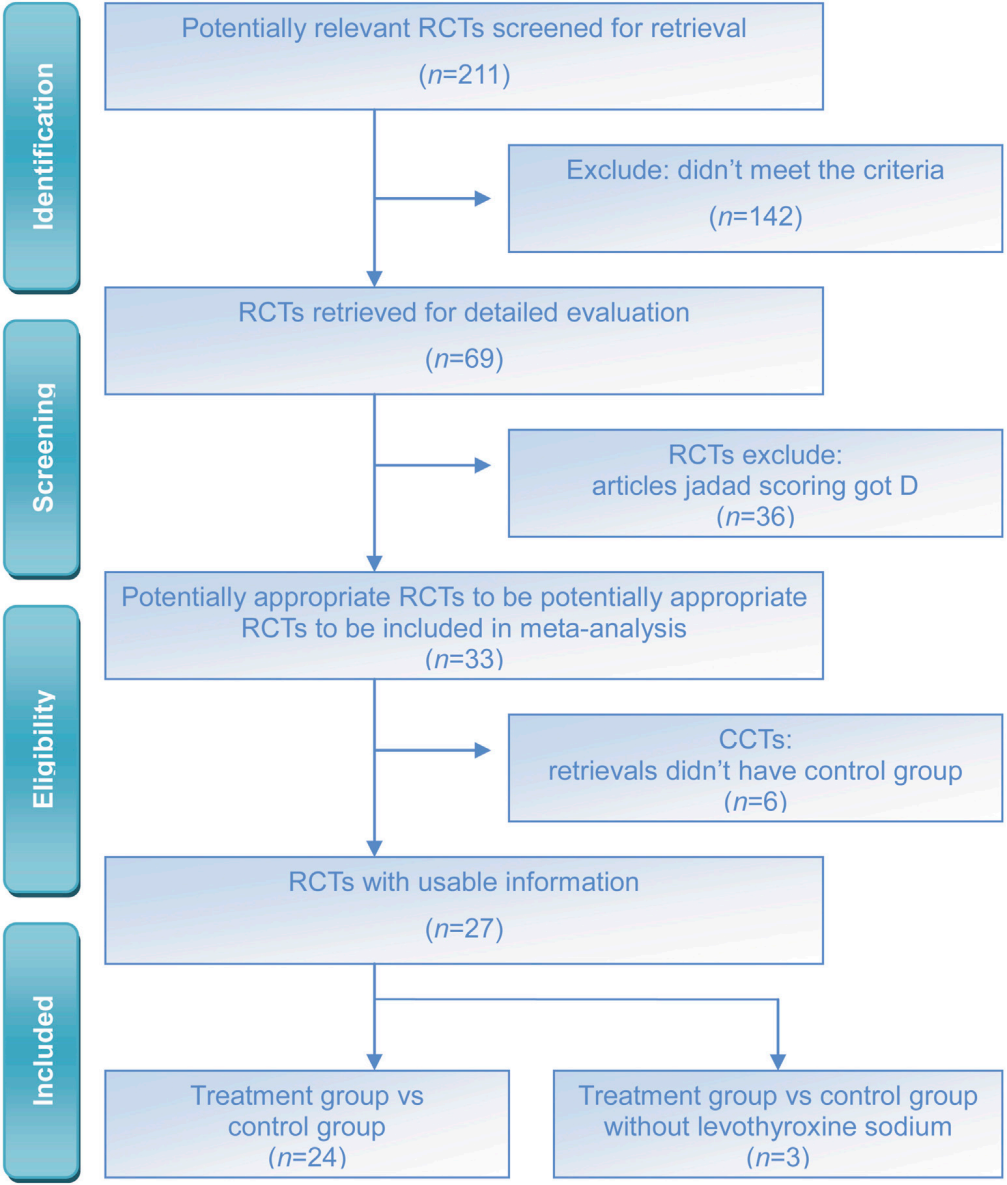
Flow diagram for identification of eligible literatures for this meta-analysis.

The literature search yielded a total of 211 published studies. The abstracts of these studies were reviewed, and subsequently, 142 studies were excluded due to a lack of controls. The 69 potentially relevant RCTs were further reviewed, of which 36 were excluded due to the low Jaded score. Finally, 33 RCTs or quasi-RCTs that met the inclusion criteria were included. 27 RCTs were included in the meta-analysis, while 6 RCTs were excluded due to the absence of a control group, but they were included in the systematic review (Figure 1). This flowchart is from the PRISMA website (https://www.prisma-statement.org/prisma-2020-flow-diagram). A total of 33 RCTs with a diagnosis of TAO were included (Table 1).

**TABLE 1:** The main characteristics of the trials.

First author	Gender (Male/Female)	Range of age and average age (years)	Tme (weeks)	Intervention		Main outcomes
				Treatment group	Control group	
Nie (2021)	92 (51/41)	31–56 (43.81 ± 5.12)	36	Thiamazole, Prednisone, Glycosides tablets	Thiamazole, Prednisone	Eyeball prominence, TSH, FT_4_, Clinical effect
Tian (2020)	44 (17/27)	20–62 (40.98 ± 10.45)	24	^99^Tc-MDP, Glycosides tablets	Glycosides tablets	Eyeball prominence, FT_3_, FT_4_, Clinical effect
Chang (2019)	86 (51/35)	29–58 (44.37 ± 10.67)	12	Thiamazole, Prednisone, Glycosides tablets	Thiamazole, Prednisone	Clinical effect
Xue and Zhang (2019)	100 (−/−)	- (−)	12	Thiamazole, Levothyroxine Sodium, Prednisone, Glycosides tablets	Thiamazole, Levothyroxine Sodium, Prednisone	Eyeball prominence, Clinical effect
Gao (2018)	90 (55/35)	30–50 (40.91 ± 10.11)	Blank	Thiamazole, Prednisone, Glycosides tablets	Thiamazole, Prednisone	Clinical effect, Quality of life score
Chi (2017)	108 (55/53)	21–49 (33.9 ± 5.3)	12	Thiamazole, Prednisone, Glycosides tablets	Thiamazole, Prednisone	Eyeball prominence, Clinical effect
Li and Ma (2017)	80 (35/45)	20–46 (32.2 ± 3.5)	12	Levothyroxine Sodium, Thiamazole, Prednisone, Glycosides tablets	Levothyroxine Sodium, Thiamazole, Prednisone	Eyeball prominence, Clinical effect
Yue et al. (2017)	53 (20/33)	-(40.2 ± 14.1)	Blank	Glycosides tablets, Levothyroxine Sodium, Methylprednisolone, Hydrochlorothiazide, Potassium chloride sustained-release tablets	Blank	Clinical effect, CAS score
Ma and Wang (2016)	80 (34/46)	20–49 (33.2 ± 1.2)	12	Thiamazole, Glycosides tablets	Thiamazole	Eyeball prominence, Clinical effect
Ao (2015)	80 (31/49)	21–45 33.2 ± 2.4	12	Levothyroxine Sodium, Thiamazole, Prednisone, Glycosides tablets	Levothyroxine Sodium, Thiamazole, Prednisone	Eyeball prominence, Clinical effect
Li (2015)	114 (41/73)	20–55 (35.70 ± 8.20)	12	Levothyroxine Sodium, Thiamazole, Prednisone, Glycosides tablets	Levothyroxine Sodium, Thiamazole, Prednisone	Eyeball prominence, Clinical effect
Luo et al. (2015)	50 (16/43)	-(46.23 ± 12.17)	16	Methylprednisolone, Prednisone, Glycosides tablets	Methylprednisolone, Prednisone	Clinical effect, CAS Score
Xv et al. (2014)	64 (29/55)	-(33.0 ± 11.5)	12	Levothyroxine Sodium, Thiamazole, Prednisone, VitD, Glycosides tablets	Levothyroxine Sodium, Thiamazole, Prednisone, VitD	Eyeball prominence, Clinical effect, Peripheral blood cytokine levels
Zhang et al. (2014)	49 (19/30)	22–47 (28.3 ± 6.9)	12	Thiamazole, Prednisone, Levothyroxine sodium, Glycosides tablets	Thiamazole, Prednisone, Levothyroxine sodium	Eyeball prominence, Clinical effect
Jiang (2013)	84 (21/63)	-(38.45 ± 1.75)	12	Thiamazole, Prednisone, Glycosides tablets	Thiamazole, Prednisone	Clinical effect, CAS Score
Cui et al. (2013)	75 (−/−)	12–70 (40.9 ± 13.1 & 37.9 ± 14.5)	12	Prednisone, Glycosides tablets	Prednisone	Clinical effect, CAS score
Liao (2012)	66 (27/39)	18–50 (32.6 ± 2.9)	12	Thiamazole, Prednisone, Glycosides tablets	Thiamazole, Prednisone	Clinical effect
Lin (2012)	122 (41/81)	18–66 (39.25)	12	Thiamazole, Prednisone, Levothyroxine sodium, Glycosides tablets	Blank	Clinical effect
Wu (2012)	60 (22/38)	19–58 (−)	12	Thiamazole, Prednisone, Levothyroxine sodium, Glycosides tablets	Blank	Clinical effect
Gou and Cheng (2012)	48 (12/36)	16–70 (43.20 ± 10.15)	12	Cetirizine tablets, Glycosides tablets	Prednisone	Clinical effect, CAS score
Zhang and Wang (2010)	98 (38/60)	19–49 (33.50)	12	Thiamazole, Prednisone, Levothyroxine sodium, Glycosides tablets	Thiamazole, Prednisone, Levothyroxine sodium	Clinical effect
He and Kong (2010)	39 (13/26)	16–65 (47.24 ± 11.16)	16	Glycosides tablets, Prednisone, ATD	Prednisone, ATD	Clinical effect, Eyeball prominence, T-cells
Zheng et al. (2010)	40 (9/31)	19–48 (35 ± 5.5)	12	ATD, Nimesulide, Glycosides tablets,^99^Tc-MDP	ATD	Clinical effect, FT_3_, FT_4_, TSH, TGAb, TPO-Ab
Wei (2009)	84 (35/49)	20–56 (35.4)	12	Thiamazole, Prednisone, Levothyroxine sodium, Glycosides tablets	Thiamazole, Prednisone, Levothyroxine sodium	Eyeball prominence, Clinical effect
Wang et al. (2009)	106 (44/62)	-(38.80)	16	Glycosides tablets,^99^Tc-MDP	Prednisone	Eyeball prominence, CAS Score, Clinical effect
Mu (2009)	61 (23/38)	17–64 (37.48)	12	Thiamazole, Prednisone, Glycosides tablets	Blank	Clinical effect
Xie et al. (2007)	60 (17/43)	17–49 (36.00 ± 5.50)	12	ATD,^99^Tc-MDP, Thiamazole	ATD	Clinical effect, FT_3_, FT_4_, TSH, TGAb, TPO-Ab
Zuo and Yang (2007)	215 (89/126)	-(48.07)	12	ATD,^99^Tc-MDP, Thiamazole, Glycosides tablets	ATD	Clinical effect, FT_3_, FT_4_, TSH, TGAb, TPO-Ab
Wang (2007)	49 (19/30)	20–53 (−)	12	Thiamazole, Glycosides tablets	Thiamazole	CAS Score
Wang et al. (2004)	48 (21/27)	30–63 (44)	16	^131^I, Glycosides tablets	^131^I, Prednisone	Clinical effect, CAS Score
Lv (2003)	22 (9/13)	20–52 (31)	12	Thiamazole, Prednisone, Levothyroxine sodium, Glycosides tablets	Blank	FT_3_, FT_4_, TSH, TGAb, TPO-Ab
Luo et al. (2002)	86 (56/30)	17–52 (34 ± 1.52)	8	Glycosides tablets	VitB_1_, VitC	Eyeball prominence
Wang (1995)	36 (14/22)	15–60 (−)	8–52	Glycosides tablets	Blank	FT_3_, FT_4_, TGAb, CIC

Notes: FT_3_, free triiodothyronine; FT_4_, free thyroxine; TGAb, thyroglobulin antibody; CIC, cycle immune complex; TSH, thyroid stimulating hormone; ATD, antithyroid drug; CAS, Score: clinical activity score.

### 3.2 Quality assessment

According to the quality evaluation standard for all included RCTs and CCTs for quality assessment and analysis. 11 RCT articles were rated as B grade. 16 articles received a C grade. The evaluation and results are presented in Tables 1, 2. Trials of poor quality (D grade) were excluded.

**TABLE 2:** The methodological quality of the trials.

First author	Year	City, Provice, Country	Random	Blind method	Baseline consistency	Fall off	Follow up	Adverse eventss	Grade
Nie (2021)	2021	Changyuan, Henan, China	Mentioned	Unclear	Consensus	Unclear	Unclear	Unclear	B
Tian (2020)	2020	Puyang, Henan, China	Mentioned	Unclear	Consensus	Unclear	Unclear	Unclear	C
Chang (2019)	2019	Tieling, Liaoning, China	Mentioned	Unclear	Consensus	Unclear	Unclear	Unclear	C
Xue and Zhang (2019)	2019	Weifang, Shandong, China	Mentioned	Unclear	Consensus	Unclear	Unclear	Unclear	C
Gao (2018)	2018	Lingyuan, Liaoning, China	Unclear	Unclear	Consensus	Unclear	Unclear	Unclear	C
Chi (2017)	2017	Xuchang, Henan, China	Mentioned	Unclear	Consensus	Unclear	Unclear	Mentioned	B
Li and Ma (2017)	2017	Yulin, Shaanxi, China	Mentioned	Unclear	Consensus	Unclear	Unclear	Unclear	C
Yue et al. (2017)	2017	Zhengzhou, Henan, China	Unclear	None	Consensus	Unclear	Unclear	Mentioned	B
Ma and Wang (2016)	2016	Bayannur, Inner Mongolia, China	Mentioned	Unclear	Consensus	Unclear	Unclear	Unclear	C
Ao (2015)	2015	Zhengzhou, Henan, China	Mentioned	Unclear	Consensus	Unclear	Unclear	Unclear	C
Li (2015)	2015	Anyang, Henan, China	Mentioned	Unclear	Consensus	Unclear	Unclear	Unclear	C
Luo et al. (2015)	2015	Enshi, Hubei, China	Mentioned	Unclear	Consensus	Unclear	Unclear	Mentioned	B
Xv et al. (2014)	2014	Jinhua, Zhejiang, China	Mentioned	Unclear	Consensus	Unclear	Unclear	Mentioned	B
Zhang et al. (2014)	2014	Shaoxing, Zhejiang, China	Mentioned	Unclear	Consensus	Unclear	Unclear	Unclear	B
Jiang (2013)	2013	Huangshi, Hubei, China	Mentioned	Unclear	Consensus	Unclear	Unclear	Unclear	C
Cui et al. (2013)	2013	Nanjing, Jiangsu, China	Mentioned	Unclear	Consensus	Unclear	Unclear	Mentioned	B
Liao (2012)	2012	Leshan, Sichuan, China	Mentioned	Unclear	Consensus	Unclear	Unclear	Unclear	B
Lin (2012)	2012	Nanning, Guangxi, China	Unclear	None	Consensus	Unclear	Unclear	Unclear	C
Wu (2012)	2012	Jiyuan, Henan, China	Unclear	None	Consensus	Unclear	Unclear	Mentioned	B
Gou and Cheng (2012)	2012	Chongqing, China	Mentioned	Unclear	Consensus	Unclear	Unclear	None	B
Zhang and Wang (2010)	2010	Pingdingshan, Henan, China	Mentioned	Unclear	Consensus	Unclear	Unclear	Unclear	B
He and Kong (2010)	2010	Guiyang, Guizhou, China	Mentioned	Unclear	Consensus	Unclear	Unclear	Mentioned	B
Zheng et al. (2010)	2010	Siping, Jilin, China	Mentioned	Unclear	Consensus	Unclear	Unclear	Unclear	C
Wei (2009)	2009	Luoyang, Henan, China	Mentioned	Unclear	Consensus	Unclear	Unclear	Unclear	B
Wang et al. (2009)	2009	Hengshui, Hebei, China	Mentioned	Unclear	Consensus	Unclear	Unclear	Mentioned	C
Mu (2009)	2009	Shangqiu, Henan, China	Unclear	None	Consensus	Unclear	Unclear	Unclear	C
Xie et al. (2007)	2007	Zhuhai, Guangdong, China	Mentioned	Unclear	Consensus	Unclear	Unclear	Mentioned	B
Zuo and Yang (2007)	2007	Kunming, Yunnan, China	Mentioned	Unclear	Consensus	Unclear	Unclear	None	C
Wang (2007)	2007	Yurao, Zhejiang, China	Unclear	Unclear	Consensus	1 case	Unclear	Mentioned	B
Wang et al. (2004)	2004	Xinxiang, Henan, China	Mentioned	Unclear	Consensus	Unclear	Mentioned	Unclear	B
Lv (2003)	2003	Ningbo, Zhejiang, China	Unclear	None	Consensus	Unclear	Unclear	Mentioned	B
Luo et al. (2002)	2002	Taiyuan, Shanxi, China	Unclear	None	Consensus	Unclear	Unclear	Unclear	C
Wang (1995)	1995	Yangzhou, Jiangsu, China	Unclear	None	Consensus	Unclear	Unclear	Unclear	C

### 3.3 Results of meta-analysis

33 RCTs or CCTs were published between 2002 and 2021 in China. There were 2,134 cases in 27 RCTs, with 1,104 cases in the treatment group and 1,030 cases in the control group. The treatment group received Tripterygium Glycosides in combination with Thiamazole, Prednisone, Levothyroxine sodium, or a combination of these medications. While the TRIO patients in the control group were treated with Thiamazole, Prednisone, Levothyroxine sodium, or a combination of these treatments. The treatment effect was categorized into four grades: cured, significantly effective, effective, and invalid.

Heterogeneity among studies was assessed using Cochran’s Q test. The *P* value (*P* < 0.01, I^2^ < 0.5) of the Q test <0.01, a random effect model was used; otherwise, a fixed effect model was used. For each model, the effect summary odds ratio (OR) and its 95% CI were calculated.

**FIGURE 2:**
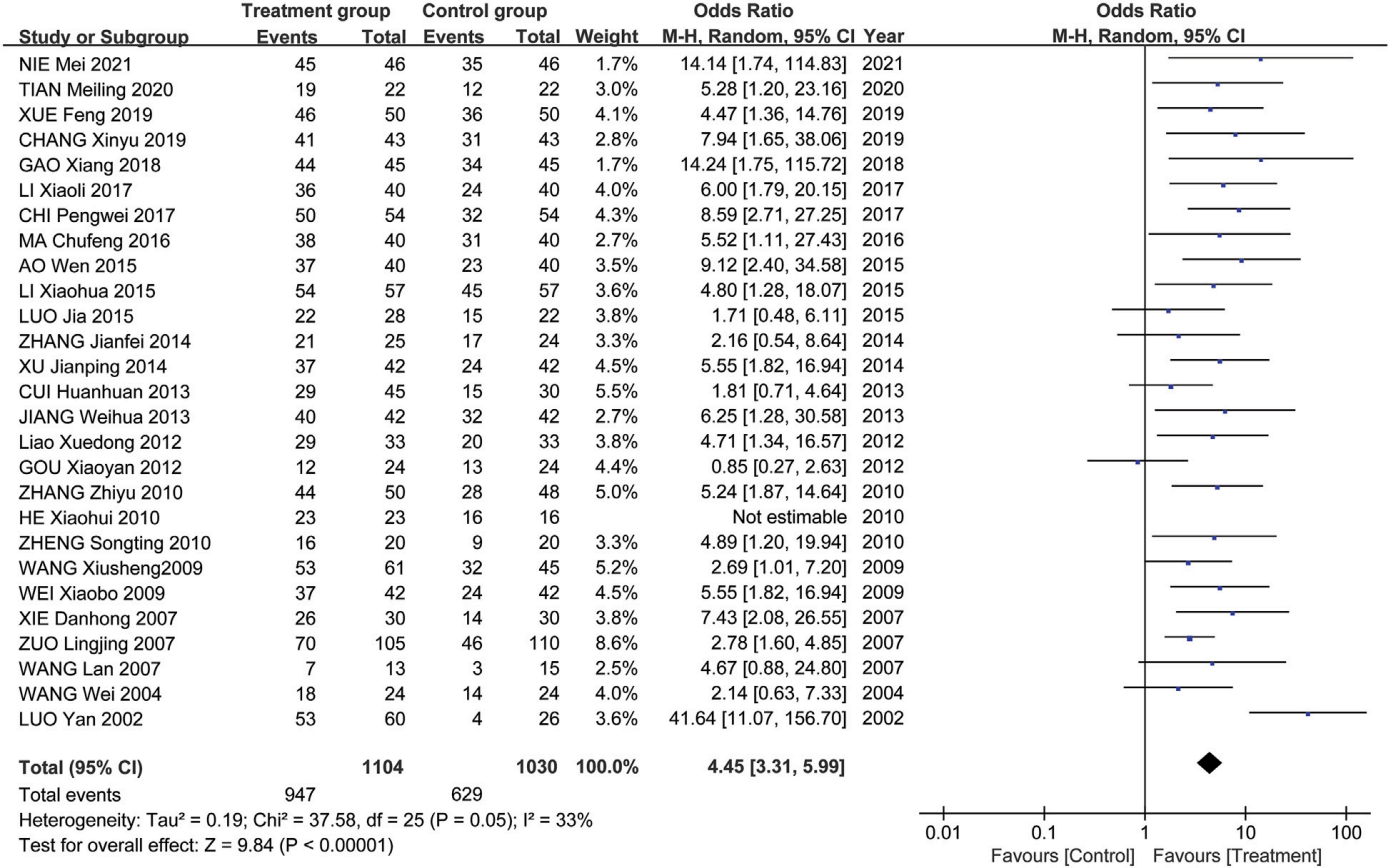
The clinical effectiveness rate.

**FIGURE 3:**
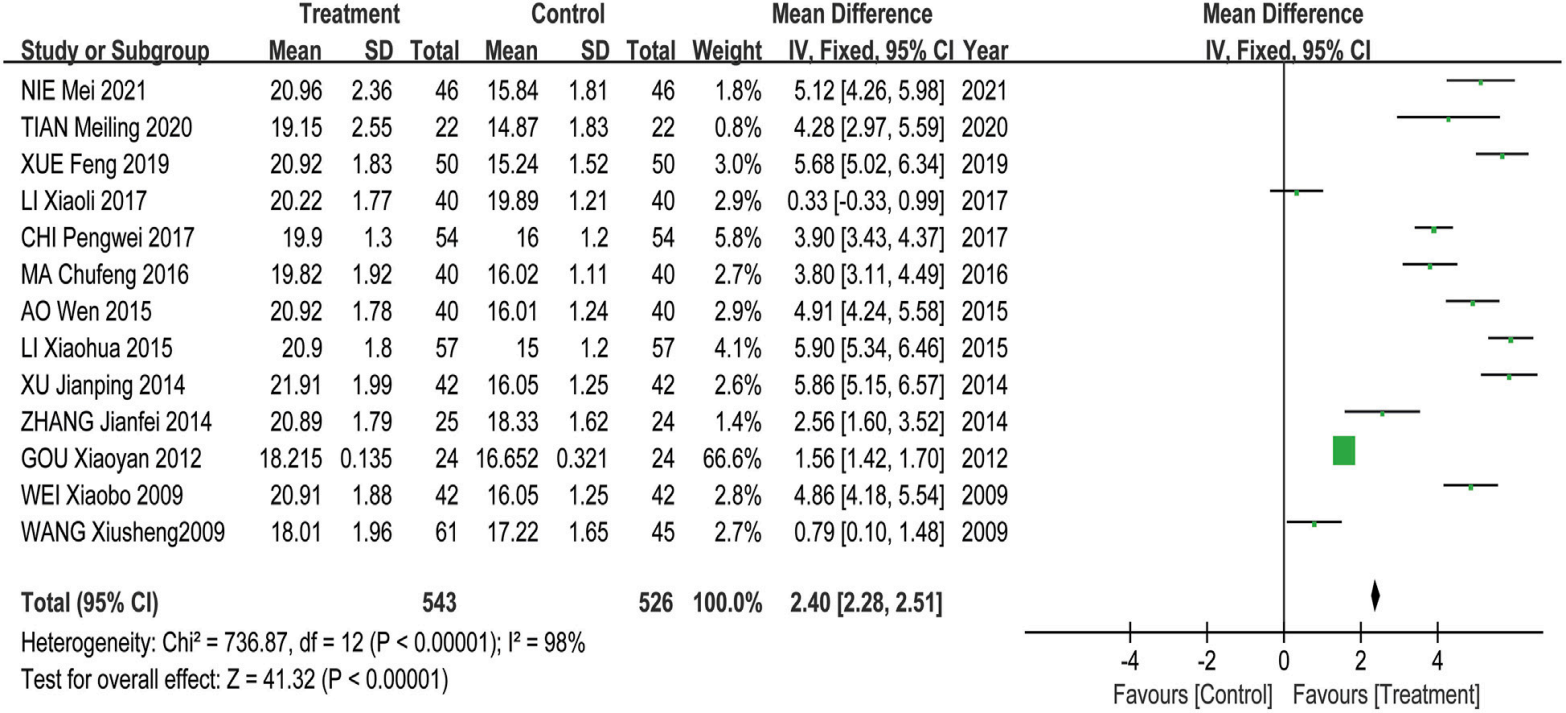
The clinical effectiveness of the eyeball prominence.

**FIGURE 4:**
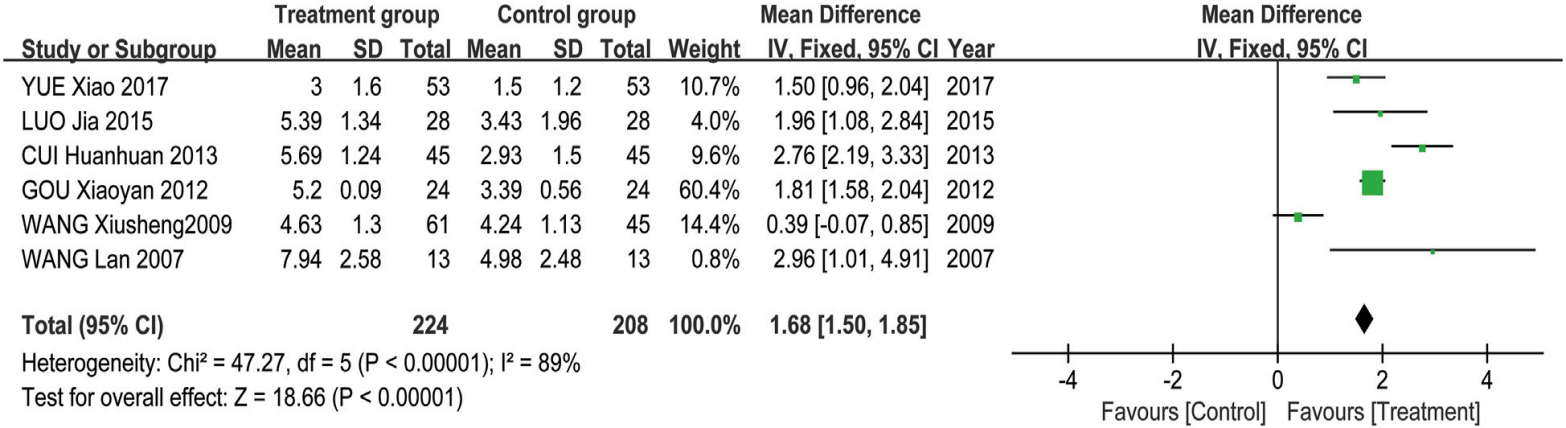
The clinical effectiveness of the eye CAS score.

Meta-analysis results showed that the overall effectiveness rate of TAO treatment in the treatment group and the control group was *P* = 0.05, I^2^ = 0.33 < 0.5 [MD = 4.45, 95% CI (3.31, 5.99), *P* < 0.00001], with the former significantly outperforming the latter. At the same time, a risk assessment was conducted for both groups in the study (Figure 2). The clinical effectiveness rate of eyeball prominence was *P* < 0.00001, I^2^ = 0.98 > 0.5 [MD = 2.40, 95% CI (2.28, 2.51), *P* < 0.00001] (Figure 3). The clinical effectiveness rate of CAS score was *P* < 0.00001, I^2^ = 0.89 > 0.5 [MD = 1.68, 95% CI (1.50, 1.85), *P* < 0.00001] (Figure 4).

**FIGURE 5:**
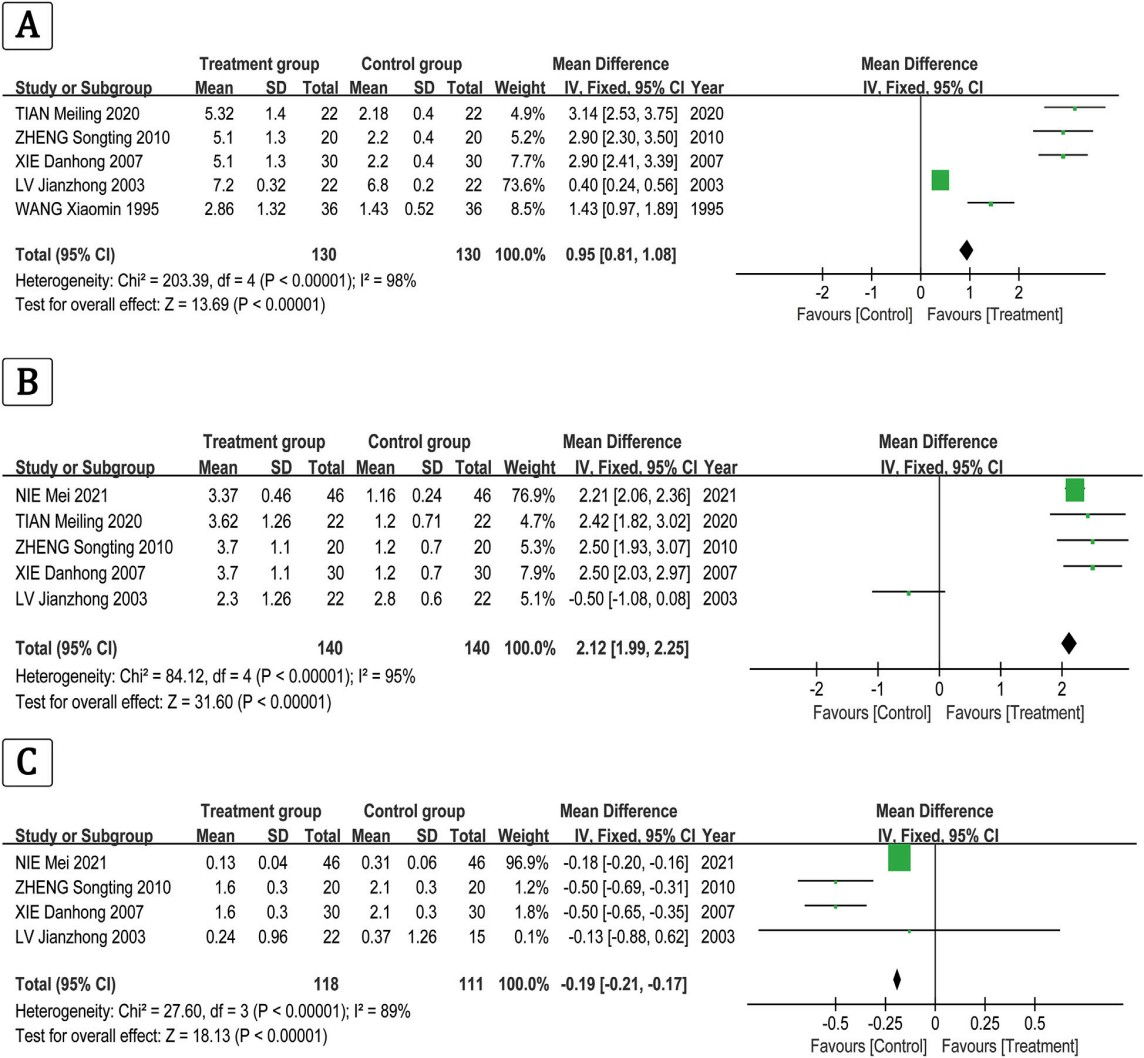
The clinical effectiveness rate of the **(A)** FT3, **(B)** FT4, and **(C)** TSH.

The clinical effectiveness rate of FT_3_ was *P* < 0.00001, I^2^ = 0.98 > 0.5 [MD = 0.95, 95% CI (0.81, 1.08), *P* < 0.00001] (Figure 5A), the clinical effectiveness rate of FT_4_ was *P* < 0.00001, I^2^ = 0.95 > 0.5 [MD = 2.12, 95% CI (1.99, 2.25), *P* < 0.00001] (Figure 5B), and the clinical effectiveness rate of TSH was *P* < 0.00001, I^2^ = 0.89 > 0.5 [MD = −0.19, 95% CI (−0.21, −0.17), *P* < 0.00001] (Figure 5C).

### 3.4 Adverse events

11 studies reported adverse events. In Chi PW’s study (Chi, 2017), 7 instances of nausea and vomiting, 3 instances of reduced appetite, and 2 instances of diarrhea were reported in the control group. In the observation group, 2 cases of nausea and vomiting, 1 case of reduced appetite, and 1 case of diarrhea were reported. In Yue X’s study (Yue et al., 2017), adverse reactions mainly occur during hormone shock and the application of somatostatin. Among them, there were 21 cases of abdominal distension, 5 cases of diarrhea, 11 cases of nausea, 5 cases of vomiting, and 3 cases of hypoglycemia, mainly during the use of somatostatin. Additionally, there were 13 cases of abnormal blood glucose: 10 cases of elevated fasting blood glucose levels and 3 cases of early morning hypoglycemia. The elevated fasting blood glucose mainly occurred during the hormone shock treatment period. After 1 week, the fasting blood glucose levels were within the normal range upon re-examination. Exciting insomnia mainly occurs during the hormone pulse therapy period, and the symptoms disappear after the completion of the pulse therapy. There were 4 cases of transient elevated blood pressure, 4 cases of abnormal liver function, and 1 case of hypokalemia. In Luo J’s study (Luo et al., 2015), 8 cases in the observation group experienced weight gain, elevated blood sugar, elevated blood pressure, and upper abdominal discomfort, respectively. In the control group, 15 cases experienced weight gain, hirsutism, epigastric discomfort, elevated blood glucose, elevated blood pressure, and elevated liver transaminase. In Xv JP’s study (Xv et al., 2014), the control group had 3 cases of weight gain, 1 case of osteoporosis, and 3 cases of peptic ulcers. In the treatment group, there were 3 cases of mild increases in serum alanine aminotransferase and 3 cases of decreased menstrual flow. In Cui HH’s study (Cui et al., 2013), 2 cases experienced mild menstrual abnormalities, 2 cases experienced stomach discomfort, 1 case had mild transaminase abnormalities, 1 case gained weight during medication, and exhibited Cushing’s face in the control group. After 1 month of medication, limb muscle stiffness occurred, but no significant changes in blood glucose were observed in all patients. There were 4 cases of mild menstrual abnormalities and 1 case of erythra in treatment group II. In Wu JT’s study (Wu, 2012), 4 cases showed a decrease in WBC, 2 cases experienced gastrointestinal reactions, and 1 case had mild liver dysfunction. All of them recovered after receiving symptomatic treatment. In He XH’s study (He and Kong, 2010), 3 cases experienced weight gain and hirsutism, while 2 cases experienced acid reflux and upper abdominal discomfort in the treatment group. In Wang XS’s study (Wang et al., 2009), there were 4 cases of short-term blood glucose elevation and 7 cases of insomnia due to excitement in the control group during the treatment process. The aforementioned side effects gradually disappeared with the decrease in hormone dosage, and no special treatment is needed. In Xie DH’s study (Xie et al., 2007), a small number of patients experienced vascular pain at the ^99^Tc MDP infusion site. The discomfort symptoms disappeared after the infusion speed was reduced in the treatment group. Some patients experienced weight gain and excessive nighttime urination after receiving low doses of dexamethasone, and no abnormalities were found in routine blood and urine tests. After the treatment, and routine blood and urine tests did not reveal any abnormalities. In Wang L’s study (Wang, 2007), 1 woman experienced amenorrhea and withdrew from the observation. However, she recovered after discontinuing the medication. In Lv JZ’s study (Lv, 2003), 21 cases experienced anorexia, nausea, vomiting, and diarrhea, and their symptoms were relieved.

### 3.5 Risk of bias within studies

Publication bias analysis was conducted on these 27 pieces of literature using a funnel plot, which resulted in a symmetric distribution. Begg’s test and Egger’s test were also conducted. Both of the *P* values were >0.05, indicating that there was no publication bias in the included trials. All the matching points were found within the 95% CI.

**FIGURE 6:**
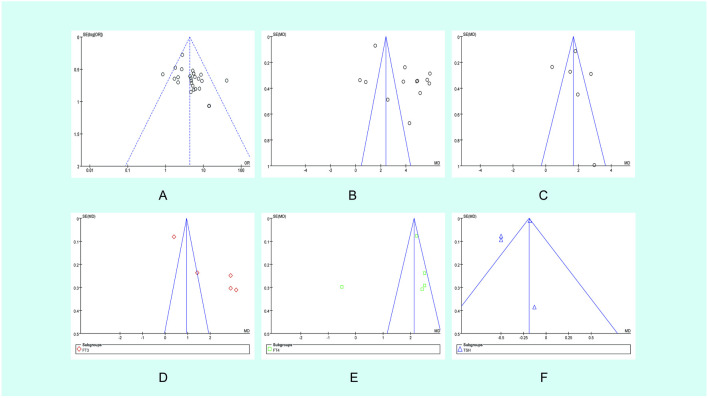
Funnel plot of the studies analyzed in the meta-analysis. **(A)** Main outcome; **(B)** Eyeball prominence; **(C)** CAS score; **(D)** FT3; **(E)** FT4; **(F)** TSH OR, odds ratio; SE, standard error.

The bias was evaluated by conducting a funnel plot analysis of the Tripterygium Glycosides treatment for TAO. The accuracy improved as the sample size increased. The amount of literature included is insufficient, scattered within the pyramid, and symmetrically distributed alongside the axis, indicating minimal bias (Figure 6A). The points corresponding to the CAS score (Figure 6C), FT_4_ (Figure 6E), and TSH (Figure 6F) in the included trials are primarily situated within the 95% CI, with a scattered distribution within the range, basically symmetrical on both sides, and presenting a funnel-shaped shape, indicating a small publication bias in the trials included. However, the eyeball prominence (Figure 6B) corresponding points were outside the range of the 95% CI, the corresponding point of FT3 (Figure 6D) is asymmetric on both sides of the axis.

## 4 Discussion

TAO is a multifactorial ocular disorder caused by thyroid disease, which often manifests as eye redness, eye pain, exophthalmos, edema, and impaired movement of periocular muscles, etc., 40%–70% of TAO patients suffer from hyperthyroidism. Currently, TAO is clinically categorized into two types: one is ocular infiltrative type, also known as endocrine ophthalmoplegia or malignant Graves’ ophthalmopathy, which accounts for 5%–10% of patients with hyperthyroidism. The other is the non-infiltrative type of ophthalmoplegia, also known as simple or benign exophthalmoplegia, which is usually caused by sympathetic stimulation of the periorbital or upper facial muscles. Ocular infiltrative TAO is an autoimmune disease caused by hyperplasia, lymphocytic infiltration and edema of the retro-ocular tissues and is influenced by a variety of factors, such as smoking, genetics and the environment (Ma and Wang, 2016; Bai, 2002).

The early pathologic changes in these diseases are the infiltration of lymphocytes and serum cells into the periocular muscles and connective tissues (Shi, 1990; Yang and Duan, 2003; Li et al., 2008). During the course of the disease, there is a buildup of collagen in the periocular muscles, which leads to fibroblasts and fat cell deposits, the presence of which is of greater significance because it indirectly confirms that the disease undergoes a longer and slower progression. The presence of fat deposits is more significant because it indirectly confirms that the disease undergoes a longer and slower progression. Current research suggests that the correlation between thyroid disease and ocular symptoms may explain the following findings: (1) edema leading to an increase in the volume of the contents of the eye sockets; (2) the production of hydrophilic glucosamines and peptidoglycans; and (3) an accumulation of adipose tissue in the eye sockets.

At the present time, the combined use of tretinoin preparations for the treatment of infiltrative TAO is widely used in the clinic. This study found that treatment with Tripterygium Glycosides may be more effective in enhancing the response rate, quality of life, and FT_3_ levels compared to treatment with Prednisone, Levothyroxine sodium, and/or Thiamazole alone. Although adverse reactions were still present in the control group, overall the rate of adverse events was lower in the observation group and the clinical benefit was much higher than in the control group. At the same time, a large body of medical evidence shows that Tripterygium Glycosides is effective in the treatment of eye protrusion in hyperthyroidism. It can inhibit cellular and humoral immunity and improve the immune status of the body. It has also been shown to inhibit the formation of self-antigens in the tissues behind the eye, thereby reducing eye protrusion (Zheng and Lv, 1982).

Tripterygium Glycosides is a traditional herbal medicine that originated from China. It has been widely used in China for the treatment of various diseases, including rheumatic diseases, skin diseases, and diabetic nephropathy. In recent years, there have been international studies conducted on Tripterygium Glycosides by renowned institutions such as Harvard University and the University of California, Los Angeles (United States), the Institute of Pharmacology and Osaka University (Japan), the Siberian Branch of the Russian Academy of Sciences and the Russian Federal Institute of Medical and Biotechnological Research (Russia), as well as the Indian Academy of Medical Sciences and the University of Delhi (India). The research conducted in these countries has mainly focused on studying the pharmacological effects, clinical applications, quality control, and other aspects of Tripterygium Glycosides.

The results of this study showed that the clinical effect of applying Tripterygium Glycosides were significantly better than those of the control group. There is a potential bias between the eyeball prominence and FT3, which may be related to the limited number of RCTs included or because the included literature did not cover all relevant indicators or only some of them were analysed. This fully explains the safety, reliability, and precise clinical efficacy of the use of Tripterygium Glycosides in the treatment of hyperthyroid eye protrusion. In addition, Tripterygium Glycosides may improve the efficacy of the basic treatment and may lead to a reduction of the drug dose or complete discontinuation of the treatment. Based on these characteristics, Tripterygium Glycosides treatment may be an ideal solution for hyperthyroidism-like herniated eye disease.

Tripterygium wilfordii, a member of the Celastraceae family, has traditionally been used in the form of a decoction. However, with the continuous advancement of modern research techniques, the chemical constituents of Tripterygium wilfordii, including raffinosides, alkaloids, and triterpenes, can now be thoroughly extracted and further developed through scientific methods. In particular, Tripterygium Glycosides tablets have gained widespread clinical use due to their convenient administration, stable efficacy, and low toxicity (Song et al., 2020; Sun et al., 2024). An increasing number of research studies have confirmed the effectiveness of Tripterygium Glycosides tablets in treating kidney diseases, rheumatism, SLE and other immune diseases, thyroid disorders, dermatological disorders and other diseases, thereby establishing a solid foundation for its clinical use (Yan et al., 2024; Xu et al., 2016; Chen et al., 2023; Hu and Gao, 2022; Liu et al., 2019; Li et al., 2023a). In recent years, numerous research teams have employed modern network pharmacology and other advanced techniques to systematically investigate the intrinsic action targets and signaling pathways of Tripterygium Glycosides tablets, as well as their toxicological effects. These studies have further validated the therapeutic efficacy of the tablets, providing crucial insights for continued in-depth research and drug development (Xiao et al., 2022; Zhu et al., 2022; Feng et al., 2022).

It is important to note that long-term use of Tripterygium Glycosides can cause some damage to various body systems. For example, long-term use of Tripterygium Glycosides at higher-than-average doses can lead to reversible liver and kidney damage, while approximately 20% of patients experience gastrointestinal reactions such as loss of appetite, nausea, vomiting, abdominal pain, diarrhea, or constipation. As for the hematopoietic system, the effects of Tripterygium Glycosides use are mainly manifested in the form of a decrease in the number of white blood cells and platelets included (Liu, 2002; Li et al., 2006). It has even been found that long-term use of Tripterygium Glycosides causes skin and mucosal reactions such as oral mucosal ulcers, dryness of the mouth and eyes, roughness and dryness of the skin, rashes, skin sclerosis, and increased melanin production, which is usually associated with the inhibition of the IL-23/IL-17 pathway (Chu et al., 2019; Qin et al., 2018). In addition, long-term use of Tripterygium Glycosides can inhibit ovarian function and cause menstrual disorders such as decreased menstrual flow or amenorrhea in women, and in men, it may lead to a decrease in sperm count or sperm motility (Li et al., 2023b; Xie et al., 2022).

Despite the inevitable problems associated with the use of Tripterygium Glycosides in the treatment of disease, its benefits in the treatment of various diseases cannot be ignored. Currently, there are several meta-analyses of interest due to the efficacy of Tripterygium Glycosides in the treatment of renal diseases, dermatological disorders, rheumatoid arthritis, and nephrotic syndrome (Li et al., 2015; Zhang and Xiang, 2013; Wang et al., 2014; Huang et al., 2015). Therefore, it is crucial to conduct a multicenter randomized, double-blind clinical trial to study the efficacy of trehalose in TAO (Sun, 2002; Wang, 2002).

## 5 Conclusion

This meta-analysis demonstrates that the experience with the treatment of TAO using Tripterygium Glycosides was promising. The existing evidence suggests that treatment with Tripterygium Glycosides may be more effective in enhancing the response rate, quality of life, and FT_3_ levels compared to treatment with Prednisone, Levothyroxine sodium, and/or Thiamazole alone.

## Data Availability

The raw data supporting the conclusions of this article will be made available by the authors, without undue reservation.

## References

[B1] Ao W. (2015). Observation on the therapeutic effect of combination therapy of Tabazole, prednisone, and Tripterygium wilfordii glycosides in patients with hyperthyroidism exophthalmos. Med. J. Chin. People’s Health 27 (13), 95–96. 10.3969/j.issn.1672-0369.2015.13.054

[B2] Bai Y. (2002). Thyroid disease-basic and clinical. Beijing: Science and Technology Literature Press, 466–476.

[B4] Chang X. Y. (2019). Effect of Tripterygium wilfordii polyglycoside combined with tapazole and prednisone on hyperthyroidism exophthalmos. Guide China Med. 17 (21), 167–168. 10.15912/j.cnki.gocm.21.129

[B5] Chen Y. Wang L. Li N. Zhou C. (2023). Tripterygium glycosides for safely controlling disease activity in systemic lupus erythematosus: a systematic review with meta-analysis and trial sequential analysis. Front. Pharmacol. 14, 1207385. 10.3389/fphar.2023.1207385 37601046 PMC10436586

[B6] Chi P. W. (2017). Clinical analysis of Tripterygium wilfordii polyglycoside combined with Thiamazole and prednisone in the treatment of hyperthyroid exophthalmos. J. North Pharm. 14 (9), 34–35.

[B7] Chu W. L. Chen S. L. Kang Z. F. Li W. Y. Liu J. (2019). Research progress on inhibition of ocular neovascularization by Chinese herbal monomers. J. Traditional Chin. Ophthalmol. 19 (2), 236–239. 10.3980/j.issn.1672-5123.2019.2.11

[B8] Cui H. H. Ye X. Z. Li Y. L. Lu B. Peng L. Xv Y. X. (2013). Clinical efficacy of mycophenolate mofetil and other Immunosuppressive drug in the treatment of thyroid associated ophthalmopathy. Chin. J. Clin. Electron. Ed. 7 (24), 11197–11200. 10.3877/cma.j.issn.1674-0785.2013.24.026

[B9] Feng Z. Fu L. Wang J. Zhu Y. He X. Zhou L. (2022). Efficacy of tripterygium glycosides (TG) in rheumatoid arthritis as a disease-modifying anti-rheumatic drug (DMARD) in combination with conventional DMARDs: a systematic review and meta-analysis of randomized controlled trials. Pharmacol. Res. 184, 106405. 10.1016/j.phrs.2022.106405 36028187

[B10] Gao X. (2018). Evaluation of the clinical efficacy of Tripterygium wilfordii polyglycoside, tapazole and prednisone in the treatment of hyperthyroid exophthalmos. Guide China Med. 16 (2), 187–188. 10.15912/j.cnki.gocm.2018.02.157

[B11] Gou X. Y. Cheng G. (2012). Clinical study on certirizine combined tripterygium glycosides for treating 24 cases of thyroid associated ophthalmopathy. China Pharm. 21 (18), 85–86.

[B12] Gu M. J. Wu W. Y. Liu C. H. Liu X. L. Li X. Liu Z. M. (2003). Efficacy of prednisone in the treatment of thyroid associated ophthalmopathy on adrenocortical function. J. Xi’an Jiaot. Univ. Med. Sci. 24 (2), 177–178.

[B13] He X. H. Kong D. M. (2010). Tripterygium wilfordii polyglycosides combined with low-dose prednisone in the treatment of 23 cases of Graves’ ophthalmopathy. New Chin. Med. 42 (8), 65–66. 10.13457/j.cnki.jncm.2010.08.078

[B14] Higgins J. P. T. Thomas J. Chandler J. Cumpston M. Li T. Page M. J. (2023). Cochrane Handbook for systematic reviews of interventions version 6.4 (Cochrane). Available at: www.training.cochrane.org/handbook. 10.1002/14651858.ED000142 PMC10284251 31643080

[B15] Hu Y. Z. Gao T. S. (2022). Meta-analysis of the efficacy and safety of combined tretinoin in the treatment of autoimmune thyroiditis. Lishizhen Med. Materia Medica Res. 33 (5), 1227–1231. 10.3969/j.issn.1008-0805.2022.05.59

[B16] Huang Q. Zeng Q. M. Zheng Y. H. Xiong J. L. (2015). Meta analysis of tripterygium wilfordii combined glucocorticoid therapy in NS patients. J. Liaoning Univ. TCM 17, 145–149. 10.13194/j.issn.1673-842x.2015.07.049

[B17] Jiang W. H. (2013). Clinical observation of Tripterygium wilfordii polyglycoside combined with tapazole and prednisone in the treatment of hyperthyroidism exophthalmos. World Health Dig. 10 (22), 178–179.

[B18] Li G. M. Li G. M. Chen L. D. (2008). A comparative observation on hyperthyroidism exophthalmos with clinical combination of Traditional Chinese and Western Medicine. Chin. J. Inf. Traditional Chin. Med. 15, 62. 10.3969/j.issn.1005-5304.2008.06.029

[B19] Li L. X. Jin R. M. Li Y. Fu S. G. Huang J. Zhu Z. L. (2006). Study on the immunosuppressive effect and safety range of multiple dosing of Tripterygium Glycosides tablets. Chin. J. New Drugs Clin. Remedies. 25, 248. 10.3969/j.issn.1007-7669.2006.02.004

[B20] Li M. Li Y. Xiang L. (2023a). Efficacy and safety of *Tripterygium* glycosides as an add-on treatment in adults with chronic urticaria:a systematic review and meta-analysis. Pharm. Biol. 61 (1), 324–336. 10.1080/13880209.2023.2169468 36694954 PMC9879204

[B21] Li M. Li Y. Xiang L. (2023b). Efficacy and safety of Tripterygium glycosides as an add-on treatment in adults with chronic urticaria: a systematic review and meta-analysis. Pharm. Biol. 61 (1), 324–336. 10.1080/13880209.2023.2169468 36694954 PMC9879204

[B22] Li W. W. Liu X. L. Wu H. Su Y. X. Li J. H. (2015). Efficacy and safety of tripterygium wilfordii Hook.F for IgA nephropathy: a meta-analysis. Chin. JEvid-based Med. 15, 206–214. 10.7507/1672-2531.20150036

[B23] Li X. H. (2015). Clinical analysis of Tripterygium wilfordii glycosides combined with Tabazolidinolone in the treatment of hyperthyroidism exophthalmos. Clin. Res. 23 (5), 60–61. 10.3969/j.issn.1004-8650.2015.05.056

[B24] Li X. L. Ma T. (2017). Clinical effect of tripterygium glycosides combined with tazobactam and prednisone on patients with hyperthyroid exophthalmos. Clin. Res. Pract. 2 (22), 75–76. 10.19347/j.cnki.2096-1413.201722037

[B25] Li X. W. (1987). Clinical observation of 50 cases of children with purpura nephritis treated with Tripterygium Glycosides tablets. Jiangsu Med. J. 12, 664–665. 10.19460/j.cnki.0253-3685.1987.12.014

[B26] Liao X. D. (2012). Clinical observation of hyperthyroidism exophthalmos treated with Tripterygium Glycosides combined with methimazole and prednisone. China Health Care Nutr. 10, 1514–1515. 10.3969/j.issn.1004-7484(x).2012.06.365

[B27] Lin Y. L. (2012). Treatment 122 cases of hyperthyroidism exophthalmos treated with Tripterygium Glycosides tablets combined with methimazole and prednisone. China J. Pharm. Econ. 7 (5), 119–120.

[B28] Liu F. (2002). Pharmacological research and clinical application of Tripterygium Glycosides tablets. Chin. Tradit. Pat. Med. 24, 385–387.

[B29] Liu X. Gao C. Liu X. Gao T. (2019). Efficacy and safety of tripterygium glycosides for Graves ophthalmopathy: a systematic review and meta-analysis. Med. Baltim. 98 (50), e18242. 10.1097/MD.0000000000018242 PMC6922466 31852090

[B30] Luo J. Huang J. Ye M. (2015). The observation of curative effect of glucocorticoids combined with Glucosida Tripterygii TOTA in the treatment of Graves’ ophthalmopathy. Pract. J. Clin. Med. 12 (5), 174–176.

[B31] Luo Y. Zheng D. W. Wang X. He Y. Q. Li D. P. (2002). Clinical observation of tripterygium wilfordii polysaccharide tablets in the treatment of thyroid related orbital lesions. China J. Chin. Ophthalmol. 12 (2), 95–97.

[B32] Lv J. Z. (2003). Prednisone and Tripterygium Glycosides tablets in the treatment of hyperthyroidism exophthalmos. Zhejiang J. Integr. Traditional Chin. West. Med. 13 (5), 299–230.

[B33] Ma C. F. Wang Y. S. (2016). Clinical efficacy of Tripterygium wilfordii glycosides combined with Methimazole in the treatment of hyperthyroid exophthalmos. World Latest Med. Inf. 16 (75), 104. 10.3969/j.issn.1671-3141.2016.75.080

[B34] Ministry of health of the people’s Liberation Army General Logistics Department (1987). Diagnosis of clinical diseases based on the improvement of the standard. Beijing: People’s military medical press, 1198–1991.

[B35] Ministry of Health PRC (1993). Guiding principle of clinical research on new drugs of Traditional Chinese Medicine. Beijing: The Medicine Science and Technology Press of China, 168. section 1.

[B36] Mu Y. D. (2009). Case analysis of Tripterygium wilfordii united tapazole prednisone in the treatment of 61 cases of thyrotoxic exophthalmoses. China Med. Her. 6 (23), 25–27.

[B37] Nie M. (2021). Efficacy evaluation of Tripterygium wilfordii polyglycoside combined with Thiamazole and prednisone acetate tablets in the treatment of hyperthyroid exophthalmos. Heilongjiang J. Traditional Chin. Med. 50 (3), 162–163.

[B38] Qin T. Y. Gao S. S. Wang W. Z. (2018). The inhibitory effect of Tripterygium wilfordii red pigment on the secretion of IL-17 by peripheral blood mononuclear cells in patients with sympathetic ophthalmitis. Chin. J. Ocular Fundus Dis. 34, 51–54. 10.3760/cma.j.issn.1005-1015.2018.01.013

[B40] Shi F. X. (1990). Observation on the effect of treating Graves eye disease with Tripterygium Glycosides tablets. New Chin. Med. 21, 472–473.

[B41] Song C. Y. Xu Y. G. Lu Y. Q. (2020). Use of Tripterygium wilfordii Hook F for immune-mediated inflammatory diseases: progress and future prospects. J. Zhejiang Univ. Sci. B 21 (4), 280–290. 10.1631/jzus.B1900607 32253838 PMC7183448

[B42] Sun H. Liu L. Wang G. Kong W. Zhong Y. Yi L. (2024). Comparison of different doses of Tripterygium glycosides treating in IgA vasculitis nephritis: a Bayesian network meta-analysis. Heliyon 10 (14), e34329. 10.1016/j.heliyon.2024.e34329 39114002 PMC11305250

[B43] Sun Z. Q. (2002). Medical statistics. Beijing: People’s Health Publishing House, 623.

[B44] Tian M. L. (2020). Therapeutic effect of Tripterygium wilfordii polyglycoside combined with technetium 99-methylenediphosphonate on thyroid associated ophthalmopathy. Pract. Clin. J. Integr. Traditional Chin. West. Med. 20 (5), 17–18. 10.13638/j.issn.1671-4040.2020.05.008

[B45] Wang J. Q. Li G. C. Zhou X. P. Ma Z. (2014). Tripterygium wilfordii extraction for treating rheumatoid arthritis: a meta-analysis. Mod. J. Integr. Traditional Chin. West. Med. 23, 1032–1036. 10.3969/j.issn.1008-8849.2014.10.003

[B46] Wang J. Y. (2002). Evidence based medicine and clinical practice. Beijing: Science press, 118–121.

[B47] Wang L. (2007). Immunosuppressive drug therapy for Graves ophthalmopathy. Zhejiang Clin. Med. J. 9, 197. 10.3969/j.issn.1008-7664.2007.02.038

[B48] Wang W. Yang B. Sun H. J. Zhou Y. L. (2004). Clinical study of ^131^I and glucoside tripterygium total tablets on Graves’ophthalmopathy. Chin. J. Nucl. Med. 24 (3), 38–39.

[B49] Wang X. M. (1995). Clinical study on the treatment of hyperthyroidism exophthalmos with Glycosides tablets. J. Jiangsu TCM 16 (10), 41–42.

[B50] Wang X. S. Li G. L. Wang Q. Ge C. J. Zhang L. (2009). Clinical study of Yunke combined with Tripterygium wilfordii glycosides in the treatment of thyroid associated ophthalmopathy. Chin. J. Pract. Ophthalmol. 27 (12), 1369–1371. 10.3760/cma.j.issn.1006-4443.2009.12.013

[B51] Webb M. S. John stone S. Morris T. J. Kennedy A. Gallagher R. Harasym N. (2010). *In vitro* and *in vivo* characterization of a combination chemotherapy formulation consisting of vinorelbine and phosphatidylserine. Eur. J. Pharm. Bio Pharm. 65 (165), 289–299. 10.1016/j.ejpb.2006.10.007 17123800

[B52] Wei X. B. (2009). The effect of Tripterygium Glycosides tablets on the treatment of hyperthyroidism exophthalmos. China Mod. Dr. 20, 97+101.

[B53] Wiersinga W. M. Prummel M. F. (2002). Grave’s ophthalmopathy: a rational approach to treatment. Trends Endocrinal Metab. 13 (7), 280–287. 10.1016/s1043-2760(02)00622-7 12163229

[B54] Wu J. T. (2012). Clinical analysis of hyperthyroidism exophthalmos treated with Tripterygium Glycosides tablets combined with methimazole and prednisone. Contemp. Med. 18 (22), 79–80. 10.3969/j.issn.1009-4393.2012.22.057

[B55] Xiao L. Xiao W. Zhan F. (2022). Targets of *Tripterygium* glycosides in systemic lupus erythematosus treatment: a network-pharmacology study. Lupus. 31 (3), 319–329. 10.1177/09612033221076725 35067081

[B56] Xie D. Li K. Ma T. Jiang H. Wang F. Huang M. (2022). Therapeutic effect and safety of tripterygium glycosides combined with western medicine on type 2 diabetic kidney disease: a meta-analysis. Clin. Ther. 44 (2), 246–256.e10. 10.1016/j.clinthera.2021.12.006 35067385

[B57] Xie D. H. Sun L. Shu X. C. Ye L. H. Shen J. Lu H. Y. (2007). Combination of technetium [^99^Tc] methylenediphosphonate, dexamasone and tripterygium glucosides in treatment of Graves ophthalmopathy. Chin. J. New Drugs Clin. Remedies 26 (12), 905–908.

[B58] Xu X. Li Q. J. Xia S. Wang M. M. Ji W. (2016). Tripterygium glycosides for treating late-onset rheumatoid arthritis: a systematic review and meta-analysis. Altern. Ther. Health Med. 22 (6), 32–39. 27866179

[B59] Xue F. Zhang W. J. (2019). Analysis of the symptom and outcome of hyperthyroidism exophthalmos treated with Tripterygium wilfordii glycosides. World Latest Med. Inf. 19 (46), 214. 10.19613/j.cnki.1671-3141.2019.46.127

[B60] Xv J. P. Xv C. Chen J. Jin Z. H. Zheng H. F. Zhu J. (2014). Graves the level of cytokines in peripheral blood of ophthalmopathy and the effect of Tripterygium wilfordii polyglycoside intervention. China J. Chin. Materia Medica 39 (3), 544–547. 24946565

[B61] Yan X. Shi J. Zhang Y. Liu J. Lin X. Yu C. (2024). Effectiveness and safety of tripterygium wilfordii poly-glycosides on glomerulonephritis: a systematic review and meta-analysis. Front. Pharmacol. 15, 1339153. 10.3389/fphar.2024.1339153 38841368 PMC11150713

[B62] Yang J. H. Duan J. G. (2003). The progress of hyperthyroidism exophthalmos with the treatment of Chinese medicine treatment. New J. Traditional Chin. Med. 35, 74. 10.3969/j.issn.0256-7415.2003.06.056

[B63] Ying J. M. (2009). Progress of Chinese medicine in treating hyperthyroid exophthalmos. J. Traditional Chin. Med. 25, 979–981. 10.3969/j.issn.1003-8914.2009.05.119

[B64] Yue X. Wang Y. Y. Yang Y. Wen S. M. Sun L. G. (2017). Clinical efficacy and safety analysis of glucocorticoids combined with somatostatin and Tripterygium wilfordii glycosides in the treatment of thyroid associated ophthalmopathy. Henan Med. Res. 26 (1), 31–33. 10.3969/j.issn.1004-437X.2017.01.012

[B65] Zhang J. F. Kong Y. Z. Pan H. Z. (2014). Clinical efficacy of the treatment of hyperthyroidism exophthalmos with Tripterygium Glycosides tablets. Chin. Rural. Health Serv. Adm. 6, 761–762. 10.19955/j.cnki.1005-5916.2014.06.057

[B66] Zhang X. M. Xiang S. M. (2013). Analysis of randomized clinical trials of oral GTT tablets in the treatment of psoriasis vulgaris. Guide China Med. 27, 392–393. 10.15912/j.cnki.gocm.2013.27.463

[B67] Zhang Z. Y. Wang J. H. (2010). Tripterygium Glycosides with tapazole and prednisone in the treatment of hyperthyroidism exophthalmos. J. Med. Forum 31 (8), 110–111.

[B68] Zheng J. R. Lv Y. (1982). Clinical and experimental studies of Tripterygium. J. Traditional Chin. Med. 23, 74–78. 10.13288/j.11-2166/r.1982.09.038

[B69] Zheng S. T. Zhang B. Mei F. (2010). Multiple enhanced immunosuppression for hyperthyroid exophthalmos: yunke, Nimesulide, Tripterygium wilfordii polyglycoside tablets for Graver ophthalmopathy. World Health Dig. 7 (23), 65–66. 10.3969/j.issn.1672-5085.2010.23.058

[B70] Zhu Y. Zhang L. Zhang X. Wu D. Chen L. Hu C. (2022). Tripterygium wilfordii glycosides ameliorates collagen-induced arthritis and aberrant lipid metabolism in rats. Front. Pharmacol. 13, 938849. 10.3389/fphar.2022.938849 36105231 PMC9465305

[B71] Zuo L. J. Yang J. S. (2007). Observation on the therapeutic effect of ^131^I combined with Tripterygium wilfordii glycosides on Graves’ ophthalmopathy. China Health Care 15 (19), 51–52.

